# Supplementary material for: Molecular classification and tumor microenvironment characteristics in pheochromocytomas
Source: eLife. 2024 Feb 26;12:RP87586. doi: 10.7554/eLife.87586 (PMC10942623; doi:10.7554/eLife.87586)
Supplement: Supplementary file 1. — (a) Somatic and Germline Mutations in 5 PCC Patients Detected by WES. (b) PASS Scores of Collected Tumor Tissues. (c) Clinical Information of 5 PCC Patients. [file elife-87586-supp1.docx]

**Supplementary File 1a: Somatic and Germline Mutations in 5 PCC Patients Detected by WES**

|  | **P1** | **P2** | **P3** | **P4** | **P5** |
| --- | --- | --- | --- | --- | --- |
| **Somatic Mutations**  **(Mutation Rates)** | JAK2 (3.2%) | ARHGEF39 (5.33%) | KMT2D (6.21%) | MST1 (2.68%) | REV3L (2.74%) |
|  | NQO1 (2.97%) | METTL4 (5.09%) | CHEK2 (1.46%) | RPA1 (1.69%) | RYR2 (2.3%) |
|  | CDH18 (2.83%) | PAK1IP1 (5.07%) | KDM6A (1.05%) | APCDD1 (1.52%) | KMT5A (2.19%) |
|  | KCNT2 (2.78%) | IGSF3 (3.2%) | CSDE1 (1.05%) | SMARCA4 (1.14%) |  |
|  | HCN1 (2.48%) | MST1 (0.85%) |  | STK11 (1.05%) |  |
|  | INSRR (2.02%) |  |  |  |  |
| **Germline Mutation**  **(Mutation Site)** | N/A | N/A | N/A | N/A | VHL (c.499C>T) |

**Supplementary File 1b: PASS Scores of Collected Tumor Tissues**

|  | **P1_T1** | **P2_T1** | **P2_T2** | **P2_T3** | **P3_T1** | **P3_T2** | **P4_T1** | **P4_T2** | **P5_T1** | **P5_T2** | **P5_T3** |
| --- | --- | --- | --- | --- | --- | --- | --- | --- | --- | --- | --- |
| **Large nest/diffuse growth >10%**  **of tumor volume** | 2 | 2 | 2 | 2 | 2 | 2 | 2 | 2 | 2 | 2 | 2 |
| **Central or confluent tumor necrosis** | 0 | 0 | 0 | 0 | 0 | 0 | 0 | 0 | 0 | 0 | 0 |
| **High cellularity** | 2 | 0 | 0 | 2 | 0 | 2 | 2 | 2 | 0 | 2 | 0 |
| **Cellular monotony** | 0 | 0 | 2 | 2 | 0 | 2 | 0 | 0 | 0 | 2 | 0 |
| **Tumor cell spindling** | 0 | 0 | 0 | 0 | 0 | 0 | 0 | 0 | 0 | 0 | 0 |
| **Mitotic figures >3/10 high power field** | 0 | 0 | 0 | 0 | 0 | 2 | 2 | 0 | 0 | 0 | 2 |
| **Atypical mitotic figures** | 0 | 0 | 0 | 0 | 0 | 0 | 0 | 0 | 0 | 0 | 0 |
| **Extension into adipose tissue** | 0 | 0 | 0 | 0 | 0 | 0 | 0 | 0 | 0 | 0 | 0 |
| **Vascular invasion** | 0 | 0 | 0 | 1 | 0 | 0 | 1 | 0 | 0 | 1 | 1 |
| **Capsular invasion** | 0 | 0 | 0 | 0 | 0 | 0 | 1 | 0 | 0 | 1 | 0 |
| **Profound nuclear pleomorphism** | 0 | 0 | 0 | 0 | 0 | 0 | 0 | 0 | 0 | 0 | 0 |
| **Nuclear hyperchromasia** | 1 | 0 | 0 | 0 | 1 | 1 | 1 | 0 | 0 | 1 | 0 |
| **Total Score** | 5 | 2 | 4 | 7 | 3 | 9 | 9 | 4 | 2 | 9 | 5 |

**Supplementary File 1c: Clinical Information of 5 PCC Patients**

|  | **Metabolism-type PCC** | | | | **Kinase-type PCC** |
| --- | --- | --- | --- | --- | --- |
|  | **P1** | **P2** | **P3** | **P5** | **P4** |
| **Tumor Size**  **(cm^3^)** | 5 × 3.8 × 3.7 | 3 × 3 × 2.5 | 5.5 × 5.5 × 4 | 3.2 × 3 × 1.7 | 6 × 5 × 4 |
| **Blood Pressure**  **(mmHg)** | 160/110 | 120/77 | 148/108 | 170/110 | 200/120 |
| **Symptom** | Hypertension  Headache  Palpitation | Asymptomatic | Hypertension  Dizziness  Weakness  Fever | Hypertension  Dizziness  Weakness | Hypertension  Headache  Palpitation  Hyperhidrosis |
| **3-methoxytyramine**  **(3-MT) (pmol/L)** | 46.2 | ＜24 | ＜24 | 37.9 | 53.7 |
| **Metanephrine**  **(MN) (pmol/L)** | 145.7 | 120.2 | 1423.6 | 62.8 | 113.4 |
| **Normetanephrine**  **(NMN) (pmol/L)** | 11928.5 | 5090.7 | 10486.6 | 2888.5 | 19215.8 |
